# Draft Genome Sequences of Clinical K1-Type Klebsiella pneumoniae Strains Isolated in Russia

**DOI:** 10.1128/MRA.01250-19

**Published:** 2020-01-02

**Authors:** Nikolay V. Volozhantsev, Angelina A. Kislichkina, Tatiana N. Mukhina, Nadezhda K. Fursova

**Affiliations:** a State Research Center for Applied Microbiology and Biotechnology, Obolensk, Moscow Region, Russian Federation; University of Southern California

## Abstract

Klebsiella pneumoniae of capsular type K1 is the most common causative agent of both health care-associated and community-acquired infections. Here, we report the draft genome sequences of 10 K1-type K. pneumoniae strains isolated from patients in an infectious disease hospital and neurosurgical intensive care unit in Russia.

## ANNOUNCEMENT

Klebsiella pneumoniae is a well-known opportunistic pathogen that causes community-acquired and health care-associated infections (1, 2). A capsular polysaccharide is the major virulence factor of K. pneumoniae (1, 3). Of the number of documented capsular types, strains of the K1 type, along with those of the K2 type, are the most virulent human pathogens (4, 5). We previously reported genome sequences of 10 strains of the K. pneumoniae K2 type, isolated from patients in an infectious disease hospital and neurosurgical intensive care unit (6). In this study, we report the genome sequences of K1-type K. pneumoniae strains isolated in the same hospitals (7).

Bacteria were grown at 37°C on nutrient medium no. 1 (Obolensk, Russia). Genomic DNA was isolated using the phenol-chloroform extraction and ethanol precipitation methods (https://fdocuments.in/download/phenol-chloroform-isoamyl-alcohol-pci-dna-isoamyl-alcohol-pci-dna-extraction). Draft genome sequencing was performed using Nextera XT DNA sample preparation kits, a MiSeq reagent kit v.3 (300 cycles), and the MiSeq platform (Illumina). For each genome, the paired reads without filtering were *de novo* assembled with Unicycler v.0.4.7 (8). Default parameters were used for all software. The resulting draft genome sizes ranged from 5.52 to 5.81 Mb, with GC contents ranging from 56.9 to 57.2%. The final assemblies were annotated with the NCBI Prokaryotic Genome Annotation Pipeline (9), resulting in the identification of total numbers of genes ranging from 6,147 to 5,453 (Table 1). Raw reads were used for multilocus sequence type (MLST) analysis with MLST v.2.0 (https://cge.cbs.dtu.dk/services/MLST/). All strains were assigned to sequence type 23.

**TABLE 1:** Strain-identifying information and basic statistics on assemblies and annotations

Strain name	Raw data SRA accession no.	GenBank accession no.	No. of reads	*N*_50_ (bp)	No. of contigs	Genome size (bp)	Total no. of genes	GC content (%)	Genome coverage (×)	Plasmid replicon type(s)	Drug resistance phenotype and predicted resistance gene(s)^*b*^								
											BLA	AMI	FQN	FOS	PHE	SUL	TRI	TET	MLS
KPS73^*a*^	SRR9208895	VKCS00000000	807,366	220,354	74	5,558,879	5,451	57.2	17	IncHI1B	*bla*_SHV-190_		*oqxA*, *oqxB*	*fosA*					
KPB1802^*a*^	SRR9208897	VKCV00000000	729,262	157,398	74	5,620,879	5,519	57.0	25	IncHI1B	*bla*_SHV-190_		*oqxA*, *oqxB*	*fosA*					
KPi1683^*a*^	SRR9208901	VKCX00000000	751,372	180,119	78	5,580,912	5,452	57.2	35	IncHI1B	*bla*_SHV-190_		*oqxA*, *oqxB*	*fosA*					
KPi3695	SRR9208904	VTRP00000000	592,094	154,881	77	5,573,189	5,453	57.1	27	IncHI1B	*bla*_SHV-190_		*oqxA*, *oqxB*	*fosA*					
KPB1493^*a*^	SRR9208896	VKCT00000000	501,624	56,694	300	5,521,123	5,669	57.0	22	IncHI1B, IncFII(K)	*bla*_SHV-190_, *bla*_CTXM15_, *bla*_TEM-1B_, *bla*_OXA-1_	*aac(6')-Ib-cr*, *aph(6)-Id*, *aph(3'')-Ib*	*aac(6')-Ib-cr*, *qnrB1*	*fosA*	*catB3*	*sul2*	*dfrA14*	*tetA*	
KPB3188	SRR9208900	VKCU00000000	717,240	105,254	202	5,614,863	5,675	57.0	31	IncHI1B, IncFII(K), IncL/M	*bla*_SHV-190_, *bla*_CTX-M-15_, *bla*_TEM-1B_, *bla*_OXA-1_, *bla*_OXA-48_	*aac(6')-Ib-cr*, *aph(6)-Id*, *aph(3'')-Ib*	*aac(6')-Ib-cr*, *oqxA*, *oqxB*, *qnrB1*	*fosA*	*catB3*	*sul2*	*dfrA14*	*tetA*	
KPB1103^*a*^	SRR9208898	VKCW00000000	660,384	97,397	151	5,594,258	5,644	57.2	29	IncHI1B, IncFII(K), IncL/M	*bla*_SHV-190_, *bla*_CTX-M-15_, *bla*_TEM-1B_, *bla*_OXA-1_, *bla*_OXA-48_	*aac(6')-Ib-cr*, *aph(6)-Id*, *aph(3'')-Ib*	*aac(6')-Ib-cr*, *oqxA*, *oqxB*, *qnrB1*	*fosA*	*catB3*	*sul2*	*dfrA14*	*tetA*	
KPB475^*a*^	SRR9208903	VTRO00000000	980,374	151,126	143	5,661,349	5,714	56.9	42	IncHI1B, IncFII(K), IncL/M	*bla*_SHV-190_, *bla*_CTX-M-15_, *bla*_TEM-1B_, *bla*_OXA-1_, *bla*_OXA-48_	*aac(6')-Ib-cr*, *aph(6)-Id*, *aph(3'')-Ib*	*aac(6')-Ib-cr*, *oqxA*, *oqxB*, *qnrB1*	*fosA*	*catB3*	*sul2*	*dfrA14*	*tetA*	
KPB470	SRR9208899	VTRN00000000	762,324	86,243	223	5,490,022	5,582	57.2	31	IncHI1B, IncFII(K), IncL/M	*bla*_SHV-190_, *bla*_CTX-M-15_, *bla*_TEM-1B_, *bla*_OXA-1_, *bla*_OXA-48_	*aac(6')-Ib-cr*, *aph(6)-Id*, *aph(3'')-Ib*	*aac(6')-Ib-cr*, *oqxA*, *oqxB*, *qnrB1*	*fosA*	*catB3*	*sul2*	*dfrA14*	*tetA*	
KPB463-13	SRR9208902	VTRQ00000000	688,138	99,224	245	5,811,379	5,966	56.9	29	IncHI1B, IncFII(K), IncL/M, Col440I, IncFIA(HI1)	*bla*_SHV-190_, *bla*_CTX-M-15_, *bla*_TEM-1B_, *bla*_OXA-1_, *bla*_OXA-48_	*aac(6')-Ib-cr*, *aph(6)-Id*, *aph(3'')-Ib*, *armA*	*aac(6')-Ib-cr*, *oqxA*, *oqxB*, *qnrB1*	*fosA*	*catB3*	*sul1*, *sul2*	*dfrA14*	*tetA*	*msr*(E), *mph*(E)

a Additional information on strain characterization is provided in a previous publication (7).

b BLA, beta-lactams; AMI, aminoglycoside; FQN, fluoroquinolone; FOS, fosfomycin; PHE, phenicol; SUL, sulphonamide; TRI, trimethoprim; TET, tetracycline; MLS, macrolide, lincosamide, and streptogramin B. Resistance phenotype was determined using a Vitek 2 Compact instrument (bioMérieux, France). ResFinder v.2.1 (14) was used to determine the presence of resistance genes.

Five types of plasmid replicons were determined in the assembled genomes using PlasmidFinder v.2.1 (10) (Table 1). All of the strains harbored a pLVPK-like virulence plasmid (11) containing an IncHI1B replicon, genes *rpmA* and/or *rmpA2* encoding regulators of the mucoid phenotype specific to hypervirulent K. pneumoniae, and siderophore gene clusters *iucABCD*, *iutA*, and *iroBCDN*. Important differences in antibiotic resistance phenotype and resistance genes between strains with different plasmid profiles were revealed (Table 1). The strains harboring only a pLVPK-like plasmid were resistant to ampicillin, fluoroquinolone, and fosfomycin due to the presence of the chromosomal genes *bla*_SHV-190_, *oqxA* and/or *oqxB*, and *fosA*, respectively. Strain KPB1493 acquired the IncFII(K) plasmid, which additionally carried genes providing resistance to aminoglycosides, phenicols, sulfonamides, trimethoprim, and tetracyclines. Strains KPB3188, KPB1103, KPB475, KPB470, and KPB463-13 harbored the IncL/M plasmid carrying the carbapenemase gene *bla*_OXA-48_ and demonstrate resistance to carbapenems. The extrachromosomal genome of strain KPB463-13 and its resistance phenotype are even more complicated because of the presence of two more plasmids, namely a cryptic plasmid, Col440I, that was detected in many extended-spectrum beta-lactamase (ESBL)-producing and carbapenem-resistant K. pneumoniae strains (12), and an IncFIA(HI1) plasmid that is possibly associated with *armA*, *sul1*, *msr*(E), and *mph*(E) genes. It is important to emphasize the identification of epidemiologically significant genes encoding the *bla*_OXA-48_ carbapenemase and the bifunctional enzyme aac(6′)-Ib-cr.

The presented diversity of the genomes in the K. pneumoniae strains reflects the important role of plasmids in the horizontal transfer of resistance genes, which is the prevalent mechanism of originating antimicrobial resistance acquisition in bacterial pathogens (13).

### Data availability.

Genome sequences were deposited in the GenBank/ENA/DDBJ databases under the accession numbers listed in Table 1.

## References

[1] Podschun R, Ullmann U 1998 *Klebsiella* spp. as nosocomial pathogens: epidemiology, taxonomy, typing methods, and pathogenicity factors. Clin Microbiol Rev 11:589–603. doi:10.1128/CMR.11.4.589. 9767057 PMC88898

[2] Shon AS, Bajwa RP, Russo TA 2013 Hypervirulent (hypermucoviscous) *Klebsiella pneumoniae*: a new and dangerous breed. Virulence 4:107–118. doi:10.4161/viru.22718. 23302790 PMC3654609

[3] Cortes G, Borrell N, de Astorza B, Gómez C, Sauleda J, Albertí S 2002 Molecular analysis of the contribution of the capsular polysaccharide and the lipopolysaccharide O side chain to the virulence of *Klebsiella pneumoniae* in a murine model of pneumonia. Infect Immun 70:2583–2590. doi:10.1128/iai.70.5.2583-2590.2002. 11953399 PMC127904

[4] Yu WL, Ko WC, Cheng KC, Lee CC, Lai CC, Chuang YC 2008 Comparison of prevalence of virulence factors for *Klebsiella pneumoniae* liver abscesses between isolates with capsular K1/K2 and non-K1/K2 serotypes. Diagn Microbiol Infect Dis 62:1–6. doi:10.1016/j.diagmicrobio.2008.04.007. 18486404

[5] Liu YM, Li BB, Zhang YY, Zhang W, Shen H, Li H, Cao B 2014 Clinical and molecular characteristics of emerging hypervirulent *Klebsiella pneumoniae* bloodstream infections in mainland China. Antimicrob Agents Chemother 58:5379–5385. doi:10.1128/AAC.02523-14. 24982067 PMC4135864

[6] Volozhantsev NV, Kislichkina AA, Lev AI, Solovieva EV, Myakinina VP, Mukhina TN, Bogun AG, Fursova NK 2018 Draft genome sequences of 10 clinical K2-type *Klebsiella pneumoniae* strains isolated in Russia. Microbiol Resour Announc 7:e01023-18. doi:10.1128/MRA.01023-18. 30533720 PMC6256651

[7] Lev AI, Astashkin EI, Kislichkina AA, Solovieva EV, Kombarova TI, Korobova OV, Ershova ON, Alexandrova IA, Malikov VE, Bogun AG, Borzilov AI, Volozhantsev NV, Svetoch EA, Fursova NK 2018 Comparative analysis of *Klebsiella pneumoniae* strains isolated in 2012–2016 that differ by antibiotic resistance genes and virulence genes profiles. Pathog Glob Health 112:142–151. doi:10.1080/20477724.2018.1460949. 29708041 PMC6056825

[8] Wick RR, Judd LM, Gorrie CL, Holt KE 2017 Unicycler: resolving bacterial genome assemblies from short and long sequencing reads. PLoS Comput Biol 13:e1005595. doi:10.1371/journal.pcbi.1005595. 28594827 PMC5481147

[9] Tatusova T, DiCuccio M, Badretdin A, Chetvernin V, Nawrocki EP, Zaslavsky L, Lomsadze A, Pruitt KD, Borodovsky M, Ostell J 2016 NCBI Prokaryotic Genome Annotation Pipeline. Nucleic Acids Res 44:6614–6624. doi:10.1093/nar/gkw569. 27342282 PMC5001611

[10] Carattoli A, Zankari E, Garcia-Fernandez A, Voldby Larsen M, Lund O, Villa L, Møller Aarestrup F, Hasman H 2014 *In silico* detection and typing of plasmids using PlasmidFinder and plasmid multilocus sequence typing. Antimicrob Agents Chemother 58:3895–3903. doi:10.1128/AAC.02412-14. 24777092 PMC4068535

[11] Chen YT, Chang HY, Lai YC, Pan CC, Tsai SF, Peng HL 2004 Sequencing and analysis of the large virulence plasmid pLVPK of *Klebsiella pneumoniae* CG43. Gene 337:189–198. doi:10.1016/j.gene.2004.05.008. 15276215

[12] Lepuschitz S, Schill S, Stoeger A, Pekard-Amenitsch S, Huhulescu S, Inreiter N, Hartl R, Kerschner H, Sorschag S, Springer B, Brisse S, Allerberger F, Mach RL, Ruppitsch W 2019 Whole genome sequencing reveals resemblance between ESBL-producing and carbapenem resistant *Klebsiella pneumoniae* isolates from Austrian rivers and clinical isolates from hospitals. Sci Total Environ 662:227–235. doi:10.1016/j.scitotenv.2019.01.179. 30690357

[13] Carattoli A 2013 Plasmids and the spread of resistance. Int J Med Microbiol 303:298–304. doi:10.1016/j.ijmm.2013.02.001. 23499304

[14] Zankari E, Hasman H, Cosentino S, Vestergaard M, Rasmussen S, Lund O, Aarestrup FM, Larsen MV 2012 Identification of acquired antimicrobial resistance genes. J Antimicrob Chemother 67:2640–2644. doi:10.1093/jac/dks261. 22782487 PMC3468078

